# Phase 1a study of the CDK4/6 inhibitor, FCN-437c, in Chinese patients with HR + /HER2- advanced breast cancer

**DOI:** 10.1007/s10637-021-01133-2

**Published:** 2021-06-09

**Authors:** Jian Zhang, Xiaojia Wang, Xian Wang, Aimin Hui, Zhuli Wu, Ling Tian, Changjiang Xu, Yuchen Yang, Wenjing Zhang, Xichun Hu

**Affiliations:** 1 Department of Medical Oncology, Fudan University Shanghai Cancer Center, 270 Dong an Road, Shanghai, 200032 China; 2 Department of Breast Medical Oncology, Cancer Hospital of the University of Chinese Academy of Sciences (Zhejiang Cancer Hospital), 1 Banshan East Street, Gongshu District, Hangzhou, 310022 China; 3 Department of Medical Oncology, Sir Run Run Shaw Hospital, 3 Qingchun East Road, Hangzhou, 310016 China; 4 Fosun Pharma USA Inc, 91 Hartwell Ave Suite 305, Lexington, MA 02421 USA; 5 Beijing Fosun Pharmaceutical Research and Development Co., Ltd, 1289 Yishan Road, Shanghai, 200233 China; 6 Avanc Pharmaceutical Co., Ltd, 55 Songshan Street, Jinzhou, 121013 China

**Keywords:** CDK4/6 inhibitor, FCN-437c, Safety, Pharmacokinetics, Antitumor activity

## Abstract

**Summary*** Purpose* This phase 1a, first-in-human study assessed the safety, maximum tolerated dose (MTD), pharmacokinetics (PK), and antitumor activity of FCN-437c, a cyclin-dependent kinase 4 and 6 (CDK4/6) inhibitor. *Methods *The study enrolled female patients with HR + /HER2- advanced breast cancer (BC) who failed standard of care therapy. A 3 + 3 dose-escalation design was utilized with a starting dose of 50 mg daily for 3 weeks on and 1 week off treatment in 28-day cycles. Patients received escalating doses of FCN-437c monotherapy (50, 100, 200, 300, and 450 mg). *Results *Seventeen patients received FCN-437c 50 mg (*n* = 3), 100 mg (*n* = 3), 200 mg (*n* = 3), 300 mg (*n* = 6), and 450 mg (*n* = 2). Two patients who received the 450-mg dose experienced dose-limiting toxicities (DLTs; grade 4 thrombocytopenia and neutropenia); no DLT was observed at any other dose level. Frequently reported treatment-emergent adverse events (TEAEs) of any grade were hematological: leukopenia (94.1%), neutropenia (88.2%), anemia (64.7%), and thrombocytopenia (47.1%). Grade 3–4 TEAEs included neutropenia (64.7%) and leukopenia (47.1%). Exposure of FCN-437c increased almost proportionally to doses ranging from 50 to 200 mg. At doses from 200 to 450 mg, there appeared to be a trend of saturation. The MTD was determined to be 300 mg. Of 15 patients with measurable disease, nine (60.0%) patients experienced stable disease; no complete or partial responses were observed. *Conclusions *These results established an acceptable safety profile for FCN-437c in patients with advanced BC, and there were no unexpected signals relative to other CDK4/6 inhibitors. (NCT04488107; July 13, 2020)

**Supplementary Information:**

The online version contains supplementary material available at 10.1007/s10637-021-01133-2.

## Introduction

Breast cancer (BC) has become the most common life-threatening malignancy in women worldwide. Although many approaches have been developed for the diagnosis and treatment of BC, the 5-year survival rate of metastatic BC remains at 27% [1]. Cyclin-dependent kinases 4 and 6 (CDK4/6) play an important role in cell proliferation and are often dysregulated in BC, particularly in hormone-receptor (HR)-positive disease [2, 3]. Cyclin D1 is a transcriptional target of the estrogen receptor (ER) and forms complexes with CDK4/6 [4]. Activation of the CDK4/6–cyclin D1 complex contributes to the hyperphosphorylation of the retinoblastoma (Rb) protein, which causes inactivation of the cell growth-inhibitory effects by releasing E2F transcription factors and the cell-cycle progression from G1 to S phase [5, 6]. Because of the essential role of this pathway in cell cycle regulation, inhibition of CDK4/6 has been regarded as a promising target for antitumor therapies. Small molecule CDK4/6 inhibitors may block tumor cell growth by binding to the ATP-binding domain of CDK4/6 kinase and dephosphorylate Rb protein, resulting in cell-cycle arrest in G1 phase [7].

The emergence of second-generation selective CDK4/6 inhibitors, which target tumors expressing CDK4/6, has resulted in meaningful prolongation of progression-free survival (PFS), compared with endocrine therapy alone [8]. To date, three orally bioavailable CDK4/6 inhibitors, palbociclib (Ibrance, PD0332991), ribociclib (Kisqali, LEE011) and abemaciclib (Verzenio, LY2834219), have received Food and Drug Administration approval for the treatment of patients with HR + /HER2- metastatic BC. These CDK4/6 inhibitors can be administered as monotherapy in heavily pretreated patients and as combination therapy with aromatase inhibitors as initial therapy or with fulvestrant after disease progression following first-line endocrine therapy. However, these treatment options are limited for patients with brain metastases. In a phase 2 study of patients with brain metastases secondary to HR + /HER2- metastatic BC who received abemaciclib, 6% achieved a confirmed objective intracranial response and 38% experienced a decrease in the sum of intracranial target lesions [9]. In addition, the intracranial clinical benefit rate (complete response [CR] + partial response [PR] + stable disease [SD] persisting for ≥ 6 months) was 25% and median PFS was 4.4 months (95% CI, 2.6–5.5) [9].

FCN-437c is an oral, second-generation, potent, and selective CDK4/6 inhibitor with no inhibitory activity against CDK1, CDK2, or CDK5. In in vitro studies, FCN-437c showed inhibitory effects on cell proliferation in human BC cell lines MCF7 and MCF/ARO, which were comparable to or greater than ribociclib and palbociclib. The synergistic antitumor effects of FCN-437c combined with fulvestrant on MCF7 cells and xenograft models were comparable to ribociclib and palbociclib: the antitumor effect of FCN-437c on MCF7/ARO xenograft models was more potent than ribociclib and palbociclib. In addition, results of nonclinical studies have shown that FCN-437c has favorable physical and pharmacokinetic (PK) properties with good penetration through the blood–brain barrier, and an acceptable toxicity profile.

## Methods

### Study design and treatment

This was a phase 1a, multicenter, open-label, single-arm dose-escalation study of FCN-437c in female patients with advanced HR + /HER2- BC. The primary objective of the study was to evaluate safety/tolerability and to determine the maximum tolerated dose (MTD) of FCN-437c as a single agent. In addition, this study evaluated the PK characteristics of FNC-437c as a single dose and continuous doses and assessed preliminary antitumor activity.

Dose escalation (50, 100, 200, 300, 450, and 600 mg daily) was conducted following a 3 + 3 study design. Patients were administered a single oral dose of FCN-437c under fasting conditions for a PK run-in of 7 days, and continuous treatment once daily for 21 days (28-day cycles). Dose-limiting toxicities (DLTs) were evaluated during the DLT observation period, including the PK run-in period (7 days) and the first cycle (28 days). The MTD was considered as the highest dose level, with no more than 33% of DLTs of assessable patients during the DLT evaluation period. All screened patients provided signed informed consent and agreed to comply with the study protocol. This study was conducted in accordance with the Declaration of Helsinki and guidelines for Good Clinical Practice as defined by the International Conference on Harmonization.

### Patients

Adult (aged ≥ 18 years) female patients with a histological or cytological confirmation of HR + /HER2- advanced BC whose disease progressed after standard therapy or for which no standard therapy was available were eligible for this study. Patients were required to have at least one measurable lesion based on Response Evaluation Criteria in Solid Tumors (RECIST) version 1.1, or only to have bone metastatic lesions, and an Eastern Cooperative Oncology Group (ECOG) performance status score of 0 or 1. Patients who recently underwent major surgery or received chemotherapy, radiotherapy, antibodies, or other investigational drugs within 28 days of enrollment, or who were previously treated with any CDK4/6 inhibitor, were excluded. Patients were also excluded if they had uncontrolled central nervous system metastases or significant cardiac dysfunction or disease.

### Safety assessments

Dose-limiting toxicities were assessed within 7 days of treatment initiation (PK run-in period) and within 28 days of treatment initiation. A DLT was defined as (1) hematological toxicities: grade 4 neutropenia lasting ≥ 3 days; grade 3 thrombocytopenia with hemorrhage; grade 3 or 4 febrile neutropenia (> 38 ℃ for 1 h or > 38.3 ℃); (2) nonhematological toxicities (alopecia excluded): grade 2 increased AST/ALT with grade 2 increased total bilirubin; QTc interval ≥ 501 ms (mean value of at least two ECGs) or QTc interval prolongation ≥ 60 ms from baseline; grade 3 nausea, vomiting and/or diarrhea, electrolyte disturbances lasting for more than 3 days, which could not be controlled or recovered to grade 1 with supportive care; and other nonspecified ≥ grade 3 nonhematological toxicities; and (3) adverse events (AEs) leading to dose suspension for more than 28 days or intolerable AEs with clinical significance as judged by the investigator. Adverse events were graded according to the National Cancer Institute’s Common Terminology Criteria for Adverse Events, v4.03.

### Pharmacokinetic assessments

Blood samples for single-dose PK evaluation were collected pre-dose and at 0.5, 1, 2, 3, 4, 6, 8, 12, 24, 48, 72, 120, and 168 h(s) post-dose. Blood samples for multidose PK evaluation were collected on Cycle 1 Day 21 (pre-dose and 0.5, 1, 2, 3, 4, 6, 8, and 12 h[s] post-dose), Day 22 (24 h post-dose), Day 23 (48 h post-dose), Day 24 (72 h post-dose) and at pre-dose of Cycle 2 Day 1 (192 h post-dose). Steady-state values were calculated by combining data collected at pre-dose of Cycle 1 Days 15, 21, and 22. Plasma samples were assayed using a validated liquid chromatography-tandem mass spectrometry assay.

The PK parameters, including area under the concentration–time curve (AUC) from dosing to the time of the last measured concentration (AUC_0-last_), AUC over the last 24-h dosing interval (AUC_0-24_), AUC to infinite time (AUC_0-∞_), maximum concentration (C_max_), time to maximum concentration (T_max_), elimination half-life (t_1/2_), oral clearance (CL/F), and accumulation ratio (R_AUC_, RC_max_), were analyzed and calculated by the noncompartmental model of Phoenix® WinNonlin 6.4 or higher version (Pharsight Corp., Certara, Princeton, NJ, USA) from the individual plasma concentration data of FCN-437c. Plasma concentration and PK parameters of FCN-437c were assessed through descriptive analysis.

### Antitumor activity assessments

Radiographic tumor assessment was performed at screening and once every 8 weeks (± 7 days) until disease progression, intolerable toxicity, or death. Assessments of antitumor activity included confirmed PR or CR, clinical benefit rate (CBR; objective response or SD for ≥ 24 weeks), and PFS per investigator assessment based on RECIST v1.1.

### Statistical methods

Sample size was estimated based on the 3 + 3 study design. All statistical analyses were performed with SAS® 9.2 or higher version (SAS Institute, Inc., Cary, NC, USA) except for the calculation of PK parameters with Phoenix® WinNonlin 6.4 or higher version (Pharsight Corp., Certara, Princeton, NJ, USA). The DLTs were summarized by counts and percentages. Safety data were summarized using descriptive statistics in patients who received at least one dose of study treatment (as-treated population). Confirmed ORR, CBR, and 95% Clopper Pearson confidence intervals were calculated. Evaluation of PFS, duration of response, and overall survival (OS) were performed using Kaplan–Meier analyses per investigator assessment.

## Results

### Patients and treatment

Between February 13, 2019 and April 15, 2020, 17 patients were enrolled at three study centers in Mainland China and received FCN-437c 50 mg (*n* = 3), 100 mg (*n* = 3), 200 mg (*n* = 3), 300 mg (*n* = 6), and 450 mg (*n* = 2). Median age was 45.0 (range, 35–67) years, and most patients (88.2%) were aged ≤ 65 years (Supplementary Table 1). All patients (100%) failed prior treatment with both hormone therapy and chemotherapy. Twelve patients discontinued treatment, all due to disease progression, and five patients remained on treatment. Five patients completed the study; four patients died and one patient completed the 1-year follow-up per protocol.

As of the data cut-off date of August 10, 2020, the median follow-up duration was 8.71 months (95% CI: 4.53–10.84). The planned median treatment duration was 112 days (range, 56–216), and the actual median treatment duration was 81 days (range, 43–157). The mean relevant dose intensity was 85.4%; 70.6% of patients received over 80% of the planned dose.

### Safety and tolerability

Two patients in the 450-mg dose group experienced DLTs and the dose was regarded as intolerable. No DLT was observed at doses of 300 mg and lower. Table 1 shows study treatment-related AEs (TRAEs) that occurred with a frequency of at least 10%. The most frequently reported hematological TRAEs were leukopenia (*n/N* = 16/17, 94.1%), neutropenia (*n/N* = 15/17, 88.2%), anemia (*n/N* = 11/17, 64.7%), and thrombocytopenia (*n/N* = 8/17, 47.1%). Frequently reported nonhematological TRAEs were prolonged electrocardiogram QT interval (*n/N* = 5/17, 29.4%), hypoalbuminemia (*n/N* = 5/17, 29.4%), and rash (*n/N* = 5/17, 29.4%). It should be noted that no grade ≥ 3 non-hematological TRAEs were reported.

**Table 1:** TRAEs Occurring in ≥ 10% of Patients (*N* = 17)

AE, *n* (%)	Grade	50 mg	100 mg	200 mg	300 mg	450 mg	Total
		(*n* = 3)	(*n* = 3)	(*n* = 3)	(*n* = 6)	(*n* = 2)	(*N* = 17)
Total	All	3 (100.0)	3 (100.0)	3 (100.0)	6 (100.0)	2 (100.0)	17 (100.0)
	Grade 3/4	0 (0.0)	0 (0.0)	3 (100.0)	6 (100.0)	2 (100.0)	11 (64.7)
Leukopenia	All	2 (66.7)	3 (100.0)	3 (100.0)	6 (100.0)	2 (100.0)	16 (94.1)
	Grade 3/4	0 (0.0)	0 (0.0)	2 (66.7)	5 (83.3)	1 (50.0)	8 (47.0)
Neutropenia	All	2 (66.7)	2 (66.7)	3 (100.0)	6 (100.0)	2 (100.0)	15 (88.2)
	Grade 3/4	0 (0.0)	0 (0.0)	3 (100.0)	6 (100.0)	2 (100.0)	11 (64.7)
Anemia	All	0 (0.0)	0 (0.0)	3 (100.0)	6 (100.0)	2 (100.0)	11 (64.7)
	Grade 3/4	0 (0.0)	0 (0.0)	0 (0.0)	0 (0.0)	0 (0.0)	0 (0.0)
Thrombocytopenia	All	0 (0.0)	0 (0.0)	1 (33.3)	5 (83.3)	2 (100.0)	8 (47.1)
	Grade 3/4	0 (0.0)	0 (0.0)	0 (0.0)	0 (0.0)	1 (50.0)	1 (5.9)
Prolonged electrocardiogram QT interval	All	1 (33.3)	0 (0.0)	2 (66.7)	2 (33.3)	0 (0.0)	5 (29.4)
	Grade 3/4	0 (0.0)	0 (0.0)	0 (0.0)	0 (0.0)	0 (0.0)	0 (0.0)
Hypoalbuminemia	All	1 (33.3)	1 (33.3)	1 (33.3)	1 (16.7)	1 (50.0)	5 (29.4)
	Grade 3/4	0 (0.0)	0 (0.0)	0 (0.0)	0 (0.0)	0 (0.0)	0 (0.0)
Rash	All	1 (33.3)	0 (0.0)	2 (66.7)	1 (16.7)	1 (50.0)	5 (29.4)
	Grade 3/4	0 (0.0)	0 (0.0)	0 (0.0)	0 (0.0)	0 (0.0)	0 (0.0)
Increased aspartate aminotransferase	All	1 (33.3)	0 (0.0)	0 (0.0)	2 (33.3)	1 (50.0)	4 (23.5)
	Grade 3/4	0 (0.0)	0 (0.0)	0 (0.0)	0 (0.0)	0 (0.0)	0 (0.0)
Increased blood bilirubin	All	0 (0.0)	1 (33.3)	2 (66.7)	0 (0.0)	1 (50.0)	4 (23.5)
	Grade 3/4	0 (0.0)	0 (0.0)	0 (0.0)	0 (0.0)	0 (0.0)	0 (0.0)
Proteinuria	All	2 (66.7)	1 (33.3)	0 (0.0)	1 (16.7)	0 (0.0)	4 (23.5)
	Grade 3/4	0 (0.0)	0 (0.0)	0 (0.0)	0 (0.0)	0 (0.0)	0 (0.0)
Asthenia	All	3 (100.0)	0 (0.0)	0 (0.0)	1 (16.7)	0 (0.0)	4 (23.5)
	Grade 3/4	0 (0.0)	0 (0.0)	0 (0.0)	0 (0.0)	0 (0.0)	0 (0.0)
Fatigue	All	0 (0.0)	0 (0.0)	1 (33.3)	2 (33.3)	1 (50.0)	4 (23.5)
	Grade 3/4	0 (0.0)	0 (0.0)	0 (0.0)	0 (0.0)	0 (0.0)	0 (0.0)
Increased alanine aminotransferase	All	1 (33.3)	0 (0.0)	0 (0.0)	1 (16.7)	1 (50.0)	3 (17.6)
	Grade 3/4	0 (0.0)	0 (0.0)	0 (0.0)	0 (0.0)	0 (0.0)	0 (0.0)
Hypercholesterolemia	All	0 (0.0)	0 (0.0)	3 (100.0)	0 (0.0)	0 (0.0)	3 (17.6)
	Grade 3/4	0 (0.0)	0 (0.0)	0 (0.0)	0 (0.0)	0 (0.0)	0 (0.0)
Hyperglycemia	All	0 (0.0)	0 (0.0)	1 (33.3)	1 (16.7)	1 (50.0)	3 (17.6)
	Grade 3/4	0 (0.0)	0 (0.0)	0 (0.0)	0 (0.0)	0 (0.0)	0 (0.0)
Hypertriglyceridemia	All	0 (0.0)	1 (33.3)	1 (33.3)	1 (16.7)	0 (0.0)	3 (17.6)
	Grade 3/4	0 (0.0)	0 (0.0)	0 (0.0)	0 (0.0)	0 (0.0)	0 (0.0)
Abdominal pain upper	All	1 (33.3)	0 (0.0)	1 (33.3)	0 (0.0)	1 (50.0)	3 (17.6)
	Grade 3/4	0 (0.0)	0 (0.0)	0 (0.0)	0 (0.0)	0 (0.0)	0 (0.0)
Diarrhea	All	0 (0.0)	1 (33.3)	0 (0.0)	1 (16.7)	1 (50.0)	3 (17.6)
	Grade 3/4	0 (0.0)	0 (0.0)	0 (0.0)	0 (0.0)	0 (0.0)	0 (0.0)
Pruritus	All	0 (0.0)	0 (0.0)	1 (33.3)	2 (33.3)	0 (0.0)	3 (17.6)
	Grade 3/4	0 (0.0)	0 (0.0)	0 (0.0)	0 (0.0)	0 (0.0)	0 (0.0)
Hyperuricemia	All	0 (0.0)	1 (33.3)	1 (33.3)	0 (0.0)	0 (0.0)	2 (11.8)
	Grade 3/4	0 (0.0)	0 (0.0)	0 (0.0)	0 (0.0)	0 (0.0)	0 (0.0)
Hypocalcemia	All	0 (0.0)	2 (66.7)	0 (0.0)	0 (0.0)	0 (0.0)	2 (11.8)
	Grade 3/4	0 (0.0)	0 (0.0)	0 (0.0)	0 (0.0)	0 (0.0)	0 (0.0)
Constipation	All	0 (0.0)	0 (0.0)	1 (33.3)	1 (16.7)	0 (0.0)	2 (11.8)
	Grade 3/4	0 (0.0)	0 (0.0)	0 (0.0)	0 (0.0)	0 (0.0)	0 (0.0)
Nausea	All	0 (0.0)	0 (0.0)	1 (33.3)	1 (16.7)	0 (0.0)	2 (11.8)
	Grade 3/4	0 (0.0)	0 (0.0)	0 (0.0)	0 (0.0)	0 (0.0)	0 (0.0)
Stomatitis	All	0 (0.0)	0 (0.0)	2 (66.7)	0 (0.0)	0 (0.0)	2 (11.8)
	Grade 3/4	0 (0.0)	0 (0.0)	0 (0.0)	0 (0.0)	0 (0.0)	0 (0.0)
Vomiting	All	0 (0.0)	0 (0.0)	1 (33.3)	1 (16.7)	0 (0.0)	2 (11.8)
	Grade 3/4	0 (0.0)	0 (0.0)	0 (0.0)	0 (0.0)	0 (0.0)	0 (0.0)
Decreased weight	All	1 (33.3)	0 (0.0)	1 (33.3)	0 (0.0)	0 (0.0)	2 (11.8)
	Grade 3/4	0 (0.0)	0 (0.0)	0 (0.0)	0 (0.0)	0 (0.0)	0 (0.0)
Increased weight	All	0 (0.0)	1 (33.3)	1 (33.3)	0 (0.0)	0 (0.0)	2 (11.8)
	Grade 3/4	0 (0.0)	0 (0.0)	0 (0.0)	0 (0.0)	0 (0.0)	0 (0.0)
Pyrexia	All	0 (0.0)	0 (0.0)	1 (33.3)	1 (16.7)	0 (0.0)	2 (11.8)
	Grade 3/4	0 (0.0)	0 (0.0)	0 (0.0)	0 (0.0)	0 (0.0)	0 (0.0)

*AE* adverse event, *TRAE* treatment-related adverse event

There were no dose adjustments or interruptions at the 50-mg or 100-mg dose level. At doses higher than 100 mg, six patients required a dose reduction (35.3%) and 10 patients required a dose interruption (58.8%) to manage hematological toxicities. No serious AEs were reported and no TEAEs led to permanent discontinuation of study treatment. There were four deaths reported in the study. One patient died as a result of respiratory failure that was considered unrelated to study treatment. The reasons for the other three deaths were unknown. All patients were in compliance of study drug (median, 100% [range, 99%-100%]).

### Pharmacokinetic characteristics

**Fig. 1:**
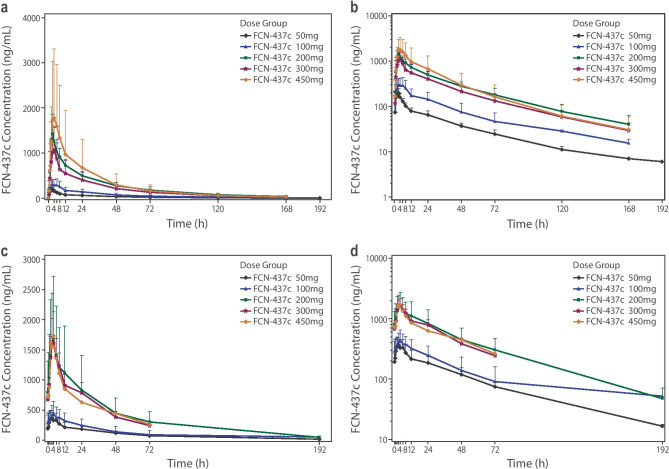
Mean plasma concentrations of FCN-437c versus time by dose level following **a** single dose (linear scale); **b** single dose (semi-log scale); **c** multiple doses (linear scale); **d** multiple doses (semi-log scale)

The plasma concentration–time data and PK profile of FCN-437c were determined in all 17 patients following a single dose on Day 1 (Fig. 1a, b, and Table 2) and multiple doses on Cycle 1 Day 21 (Fig. 1c, d, and Table 2). The correlation of exposure and dose after multidose administration was analyzed (Supplementary Table 2).

**Table 2:** Pharmacokinetic Parameters of FCN-437c

Mean ± SD	50 mg	100 mg	200 mg	300 mg	450 mg
Day 1: Following single dose					
C_max_ (ng/mL)	240 ± 78.9	316 ± 121	1477 ± 376	1092 ± 489	1839
T_max_ (h)^a^	3.00 (1.00 ~ 3.00)	3.00 (1.00 ~ 4.00)	3.00 (2.00 ~ 3.00)	3.50 (2.00 ~ 4.00)	3.50 (3.00 ~ 4.00)
AUC_0-∞_ (ng∙h/mL)	6115 ± 1015	12,003 ± 6242	45,146 ± 12,812	34,648 ± 13,642	51,591
t_1/2_ (h)	50.6 ± 3.42	40.9 ± 14.0	42.5 ± 3.18	43.6 ± 9.50	37.1
MRT (h)	58.7 ± 9.06	48.7 ± 13.3	50.1 ± 6.96	51.9 ± 15.0	40.2
Vd/F (mL/kg)	9893 ± 1057	8674 ± 2537	4020 ± 1079	9765 ± 3341	13,210
Cl/F (mL/h/kg)	136 ± 11.3	173 ± 118	66.3 ± 20.7	158 ± 62.4	248
Cycle 1 Day 21: Following multiple doses					
C_max_ (ng/mL)	378 ± 122	469 ± 179	1723 ± 983	1653 ± 457	1730
T_max_ (h)^a^	3.00 (2.00 ~ 3.00)	4.00 (3.00 ~ 8.00)	4.00 (3.00 ~ 4.00)	4.00 (3.00 ~ 4.00)	4
AUC_0-∞_ (ng∙h/mL)	18,568 ± 4437	22,566 ± 16,691	71,273 ± 35,143	56,592 ± 12,149	58,803
t_1/2_ (h)	52.1 ± 8.20	38.8 ± 24.3	45.1 ± 14.1	27.9 ± 2.67	36.6
R_AUC_	3.12 ± 1.00	1.88 ± 0.701	1.61 ± 0.819	1.59 ± 0.456	3.04
Vd/F (mL/kg)	3531 ± 1312	4222 ± 894	3301 ± 2243	3408 ± 704	6858
Cl/F (mL/h/kg)	46.0 ± 11.7	98.7 ± 58.6	47.6 ± 26.1	86.1 ± 24.4	130
R_Cmax_	1.63 ± 0.458	1.58 ± 0.525	1.24 ± 0.838	1.50 ± 0.643	2.12
AUC_0-24_ (ng∙h/mL)	5889 ± 2138	7871 ± 3109	27,253 ± 17,592	25,098 ± 5019	23,615
C_av-ss 24 h_(ng/mL)	245 ± 89.1	328 ± 130	1136 ± 733	1046 ± 209	984
DF _24h_^b^	0.802 ± 0.222	0.712 ± 0.245	0.863 ± 0.236	0.820 ± 0.194	1.12

*AUC*_0-__∞_ area under the curve from 0 h after administration to infinite time point, *AUC*_0-24_ area under the curve from 0 h after administration to 24 h, *C*_av-ss 24 h_ average steady-state concentration from 0 h after administration to 24 h, *Cl/F* apparent volume of the central compartment cleared of drug per unit time, *C*_max_ maximum concentration, *DF*_24 h_ degree of steady-state concentration fluctuation from 0 h after administration to 24 h, *MRT* mean residence time, *R*_AUC_ ratio of AUC_0-__∞_, *RC*_max_ ratio of maximum concentration, *t*_1/2_ terminal half-life, *T*_max_ time to C_max_, *Vd/F* apparent volume of distribution per unit of body weight

^a^T_max_ (h) is presented as median (range)

^b^DF _24 h_ = 100% × (C_max_-C_min_)/C_av-ss 24 h_

#### On single-dose administration

FCN-437c was absorbed rapidly with a median T_max_ ranging from 3.0 to 3.5 h. Mean t_1/2_ ranged from 37.1 to 50.6 h. Mean apparent volume of distribution per unit of body weight ranged from 4020 to 13,210 mL/Kg (Table 2). The exposure (C_max_ and AUC_0-∞_) increased almost in proportion with doses ranging from 50 to 450 mg.

#### On multidose administration

Mean C_max_, average steady-state concentration from 0 h after administration to 24 h (C_av-ss 24 h_), AUC_0-∞_, and AUC_0-24_ ranged from 378–1723 ng/ml, 245–1136 ng/ml, 18,568–71,273 ng∙h/mL, and 5889–27,253 ng∙h/mL, respectively, for doses ranging from 50 to 200 mg. After repeated administration, FCN-437c accumulated in the human body. Mean accumulation ratio for AUC_0-∞_ (R_AUC_) and C_max_ (R_Cmax_) ranged from 1.59–3.12 and 1.24–1.63, respectively (Table 2). The exposure increased almost in proportion with dose following multidose administration from 50 to 200 mg. After multidose administration of the doses ranging from 200 to 450 mg, there appeared to be a trend of saturation.

### Antitumor activity

**Fig. 2:**
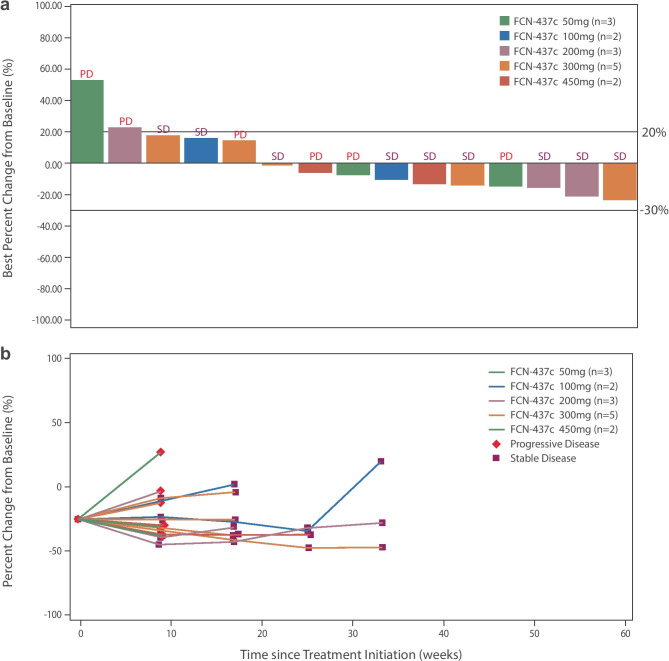
**a** Waterfall plot and **b** spider plot of best percentage change from baseline in sum of diameters by dose level

Among 15 patients with post-baseline assessments, nine patients experienced SD (60%) (Table 3 and Fig. 2a). At the time of data cut-off, one patient had maintained SD for over 6 months (Fig. 2b); the CBR was 6.7%. Median PFS was 3.91 months (95% CI: 2.07–7.62), and median OS had not been reached at the time of analysis.

**Table 3:** Clinical Antitumor Activity of Single Agent FCN-437c

	Total (*N* = 15)
Best overall response, *n* (%)	
Complete response	0 (0.0%)
Partial response	0 (0.0%)
Stable disease	9 (60.0%)
Progressive disease	6 (40.0%)
Not evaluable	0 (0.0%)
ORR, % (95% CI)	0.0% (0.00%-21.80%)
CBR, % (95% CI)^a^	6.7% (0.17%-31.95%)
Median PFS, months (95% CI)	3.91 (2.07–7.62)
Median OS, months (95% CI)	NR (7.98-NA)

*CBR* clinical benefit rate, *NA* not applicable, *NR* not reached, *ORR* objective response rate, *OS* overall survival, *PFS* progression-free survival

^a^CBR is defined as the proportion of patients with confirmed complete response + partial response + stable disease lasting for ≥ 6 months

## Discussion

This phase 1a dose-escalation study enrolled adult female patients with advanced/metastatic HR + /HER2- BC to evaluate the safety and tolerability of FCN-437c, an oral CDK4/6 inhibitor. Preliminary data from the 17 patients who participated in the study demonstrated an acceptable safety profile for FCN-437c. Most reported AEs were hematologic in nature and included leukopenia, neutropenia, and anemia, which were manageable and reversible with supportive care and were strongly correlated with dose level. Two patients in the 450-mg dose group experienced DLTs in the third week after the initiation of the first cycle in the continuous dose treatment period: one grade 4 thrombocytopenia that lasted for 11 days and one grade 4 leukopenia that lasted for 7 days. No DLTs were reported in patients who received doses of 300 mg and lower. Neutropenia and leukopenia were the most frequently reported grade 3 and 4 AEs that occurred in patients who received FCN-437c in doses of 200 to 450 mg and were the AEs that most commonly led to dose reduction and dose interruption. In general, the toxicity profile of FCN-437c is similar to that established for palbociclib and ribociclib [10, 11]. For example, hematologic toxicities are the most commonly reported AEs of any grade among patients treated with palbociclib and ribociclib and the most frequently reported grade 3/4 AE is neutropenia [10, 11].

Patients who received FCN-437c also experienced mild-to-moderate nonhematologic AEs, all of which were grade 1 or 2. Prolonged electrocardiogram QT interval, hypoalbuminemia, and rash were the most commonly reported nonhematological toxicities. Of note, grade 1 or grade 2 QTcF prolongation was reported in five patients (29.4%). In contrast, a high frequency of prolonged QTc interval has been reported in patients treated with ribociclib [12]. Results from the MONALEESA-7 study showed an increase of more than 60 ms from baseline in the QTcF interval in 32 of 335 (10%) patients in the ribociclib group; this AE led to dose interruptions or dose reductions in 13 (4%) patients [12]. Abemaciclib, however, is associated with lower rates of hematological toxicities, such as grade 3/4 neutropenia (21.1%), and higher rates of diarrhea, nausea, fatigue, and increased creatinine [13, 14]; none of these AEs were commonly reported in the current study.

Results of the current study showed that when administered as a single dose, the exposure (C_max_ and AUC_0-∞_) of FCN-437c increased almost in proportion with doses from 50 to 450 mg. When FCN-437c was administered in multiple doses, the exposure (C_max_, AUC_0-∞_, AUC_0-24_, C_av-ss 24 h_) also increased almost in proportion with doses from 50 to 200 mg. The concentration of FCN-437c reached steady state at Cycle 1 Day 15. As the FCN-437c dose increased from 50 to 200 mg, the average trough concentration increased in nearly equal proportion after multidose administration. At multidose administration of higher doses (200 to 450 mg), there appeared to be a trend of saturation. Elimination characteristics of FCN-437c were similar between single doses and multiple doses at all dose levels. After repeated administration, FCN-437c accumulated in the human body. Mean accumulation ratio for AUC_0-∞_ and C_max_ ranged from 1.59 to 3.12 and from 1.24 to 1.63, respectively. Results of a multi-disciplinary review of the PK characteristics of ribociclib from the CLEE011X2101 study showed that there is a large variation between individuals (C_max_ range, 859–5860 ng/ml; AUC_0-24 h_ range, 9960–89,600 ng∙h/ml) [15, 16]. In addition, FCN-437c exposure appears to be higher than ribociclib exposure. Following a single dose, the mean C_max_ of FCN-437c 200 mg (1477 ± 376 ng/ml) was higher than the mean C_max_ of ribociclib 600 mg (geometric mean, 933 [340–3200] ng∙h/ml). Following multiple doses, the FCN-437c C_max_ (1723 ± 983 ng/ml) and AUC_0-24_ (27,253 ± 17,592 ng∙h/ml) in the 200-mg dose group were similar to ribociclib’s C_max_ (geometric mean, 1940 [range, 859–5860] ng/ml) and AUC_0-24 h_ (geometric mean, 26,600 [range, 9960–89600] ng∙h/ml) in the 600-mg dose group.

Results of the current study established the MTD of FCN-437c as 300 mg once daily based on the occurrence of grade 3 or 4 hematologic AEs and the rate of dose interruptions and adjustments that occurred with this dose. In addition, there appears to be a trend of the exposure saturation from 200 to 450 mg, thus the recommended phase 2 dose of FCN-437c was proposed as 200 mg.

Results of studies that assessed the three marketed CDK4/6 inhibitors showed that combination therapies with endocrine agents (aromatase inhibitors or fulvestrant) significantly improved PFS and OS over the placebo plus endocrine agents in patients with HR + /HER2- metastatic BC in first- and second-line settings, regardless of endocrine therapy strategies, treatment lines, number of metastatic sites, or menopausal status [17, 18]. However, minor antitumor activity was demonstrated with single-agent palbociclib or ribociclib. Few patients achieved PRs and approximately 30% of patients experienced SD in phase 1 trials [19–21]. In the phase 2 MONARCH-1 study, abemaciclib alone showed a confirmed ORR of 19.7% and a CBR of 42.4% among 132 heavily pretreated patients; median PFS was 6.0 months and median OS was 17.7 months [22]. Based on these results, abemaciclib has become the only CDK4/6 inhibitor approved as monotherapy for the treatment of patients with HR + /HER2- advanced BC whose disease progressed following prior endocrine therapy and chemotherapy.

Similar to palbociclib and ribociclib, FCN-437c as monotherapy showed modest antitumor activity; however, 60% of the patients experienced durable SD, which suggests potential for improved antitumor activity when combined with hormone therapy. Results of the current study warrant further exploration of FCN-437c combination therapy in phase 2 studies.

In conclusion, this study showed that single-agent FCN-437c administered on a 3/1 schedule has a favorable safety profile. Most toxicities were hematologic in nature and were generally tolerable and manageable. FCN-437c 200 mg is the proposed recommended phase 2 dose based on the comprehensive assessment of safety and PK in the current study. Preliminary results showing that 60% of patients who received single-agent FCN-437c experienced SD warrant further exploration in clinical trials, particularly to evaluate FCN-437c in combination with other endocrine therapies. Currently, a phase 2 trial of FCN-437c combined with letrozole or fulvestrant in female patients with advanced/metastatic HR + /HER2- BC is ongoing.

## Supplementary Information

Below is the link to the electronic supplementary material.

Supplementary file1 (DOCX 18 KB)
