# Supplementary material for: Phase 1a study of the CDK4/6 inhibitor, FCN-437c, in Chinese patients with HR + /HER2- advanced breast cancer
Source: Invest New Drugs. 2021 Jun 9;39(6):1549–58. doi: 10.1007/s10637-021-01133-2 (PMC8541945; doi:10.1007/s10637-021-01133-2)
Supplement: Supplementary file 1 — Supplementary file1 (DOCX 18 KB) [file 10637_2021_1133_MOESM1_ESM.docx]

**Supplementary Materials**

**Supplementary Table 1** **Patient Characteristics and Disposition at Baseline**

|  | **Total Population (*N*=17)** |
| --- | --- |
| Median age, years (range) | 45.0 (35-67) |
| Median weight, kg (range) | 65.50 (50.00-80.50) |
| ECOG performance status, n (%) |  |
| 0 | 3 (17.6%) |
| 1 | 14 (82.4%) |
| Prior antitumor therapies, n (%) |  |
| Any | 17 (100.0%) |
| Surgery | 14 (82.4%) |
| Chemotherapy | 17 (100.0%) |
| Radiotherapy | 10 (58.8%) |
| Endocrine therapy | 17 (100.0%) |
| Targeted therapy | 1 (5.9%) |

*ECOG* Eastern Cooperative Oncology Group

**Supplementary Table 2** **Correlation of Exposure and Dose After Multidose Administration**

| **Dose** | **DR** | **C_max_** | **ER** | **AUC**  **_0-∞_** | **ER** | **AUC_0-24_** | **ER** | **C_av-ss0-24_** | **ER** | **C_trough_** | **ER** |
| --- | --- | --- | --- | --- | --- | --- | --- | --- | --- | --- | --- |
|  |  |  |  |  |  |  |  |  |  |  |  |
| 50 | 1 | 378 | 1 | 18568 | 1 | 5889 | 1 | 245 | 1 | 188 | 1 |
| 100 | 2 | 469 | 1.24 | 22566 | 1.22 | 7821 | 1.33 | 328 | 1.34 | 299 | 1.59 |
| 200 | 4 | 1723 | 4.56 | 71273 | 3.84 | 27253 | 4.63 | 1136 | 4.64 | 812 | 4.33 |
| 300 | 6 | 1870 | 4.95 | 56592 | 3.05 | 25098 | 4.26 | 1046 | 4.27 | 730 | 3.89 |
| 450 | 9 | 1730 | 4.58 | 58803 | 3.17 | 23615 | 4.01 | 984 | 4.02 | 661 | 3.52 |

The exposure (C_max_, AUC_0-∞_, AUC_0-24_, C_av-ss 24h_, trough concentration) of 50 mg was set to 1 and the exposure of the other doses was compared to the exposure of 50 mg. The dose of 50 mg was set to 1 and the other doses were compared to 50 mg.

*AUC_0-∞_* area under the curve from 0 hour after administration to infinite time point, *AUC_0-24_* area under the curve from 0 hour after administration to 24 hours, *C_av-ss 24h_* average steady-state concentration from 0 hour after administration to 24 hours, *C_max_* maximum concentration, *C_trough_* trough concentration, *DR* dose ratio, *ER* exposure ratio
